# Using a low melting solvent mixture to extract value from wood biomass

**DOI:** 10.1038/srep32420

**Published:** 2016-09-07

**Authors:** Jaakko Hiltunen, Lauri Kuutti, Stella Rovio, Eini Puhakka, Tommi Virtanen, Taina Ohra-Aho, Sauli Vuoti

**Affiliations:** 1 VTT Technical Research Centre of Finland LTD, Biologinkuja 7, P.O. Box 1000, FI-02044 VTT, Finland

## Abstract

Green chemistry, sustainability and eco-efficiency are guiding the development of the next generation of industrial chemical processes. The use of non-edible lignocellulosic biomass as a source of chemicals and fuels has recently raised interest due to the need for an alternative to fossil resources. Valorisation mainly focuses on cellulose, which has been used for various industrial scale applications for decades. However, creating an economically more viable value chain would require the exploitation of the other main components, hemicellulose and lignin. Here, we present a new low melting mixture composition based in boric acid and choline chloride, and demonstrate its efficiency in the fractionation of wood-based biomass for the production of non-condensed lignin, suitable for further use in the search for sustainable industrial applications, and for the selective conversion of hemicelluloses into valuable platform chemicals.

The goal of modern biorefineries is valorisation of the entire lignocellulosic biomass, in an attempt to offset processing costs by providing a wide range of products of both high and low value^1^,^2^. Although lignin is the second-most abundant terrestrial polymer after cellulose, around 60% more lignin is generated by the pulp and paper industry than is needed to meet internal energy use through combustion^3^. Lignin-based products with a higher value than that derived from their use as an energy source will prove vital to the economy of future biorefineries. Lignin has major potential as an aromatic resource for bulk chemical and fuel production^4^,^5^,^6^,^7^. Advantages have been noted in the use of lignin as such or following chemical modification in the form of composites, dispersions and especially carbon fibres^8^,^9^ and as a renewable source of aromatic chemicals6. However, attempts to use lignin in carbon fibres or composites and plastics have not yielded commercially favourable results. Poor mechanical properties, resulting from the presence of branched high molecular weight fractions formed by condensation reactions in the Kraft pulping process, have hindered the large-scale use of lignin in carbon fibres^10^. Process impurities such as sulphur, variable molecular weights and poor processibility have been listed as possible reasons for failure in composite products^3^. The effective chemical modification or depolymerisation of lignin requires the preservation of a maximum amount of accessible aliphatic hydroxyl groups naturally present in lignin^8^,^10^. Industrially feasible extraction of lignin while preserving its natural structure has therefore become the goal of novel fractionation processes.

Deep eutectic solvents (DESs), also often described as low melting mixtures, have recently emerged as a new potential breakthrough technology for the conversion of biomass into valuable platform chemicals and polysaccharide components^11^,^12^. DESs were first presented by Abbott *et al*.^13^. In principle, DESs are a mixture of solids at different molar ratios, which ideally at room temperature produce a liquid with solvent properties. This enables the use of the chemicals as such without additional solvents and most DESs exhibit unpredicted solvent properties. DESs consist of a hydrogen bond acceptor, typically a quaternary ammonium halide salt, combined with a hydrogen bond donor such as a carboxylic acid, amino acid, alcohol, amine or carbohydrate4. A well-known example is the combination of choline chloride (m.p. 302 °C) with urea (m.p. 132 °C), to form a DES with a melting point of 12 °C^13^. DESs can be chemically inert with respect to water and i.e. enable the use of enzymes that are in many cases completely incompatible with organic solvents and ionic liquids^14^. A DES consisting of choline chloride – lysine and water has been used successfully for sugar cane bagasse pretreatment prior to enzymatic hydrolysis^15^. The authors reported that 61.5% of lignin was extracted using this DES composition and 44.9–63.3% removal of lignin was achieved using other choline chloride – amino acid DESs. Similar results were achieved by Kumar *et al*.^16^ while treating rice straw. However, the DESs compositions presented in these publications did not provide fractionation efficiency in the case of wheat straw^17^.

## Results

In our search for the optimum low melting mixture composition, the aim was to provide high fractionation efficiency for the components of wood, without causing the degradation or condensation of major lignocellulosic components. Abbott *et al*.^13^ showed how the physical properties of DESs, such as melting point, viscosity and Lewis acidity could be tuned by selection of the components. Boric acid is a Lewis acid capable of forming labile intermediates with many polysaccharides^18^. Because boric acid’s acidity is explained by electron pair acceptance rather than proton donation, it is categorised as a monoprotic acid, not a triprotic as could be expected. In boric acid, the boron atom possesses a formally vacant p-orbital; it is bound to highly electronegative oxygen atoms, which withdraw electron density and create a partial positive (δ+) charge. In addition, boric acid has a planar geometry that makes it quite sterically accessible to approaching nucleophiles. This enables the formation of covalent interactions with electron donating species. Boric acid is categorised as a weak Lewis acid and its use has been reported to be beneficial in the protection of lignin by forming esters with its hydroxyl groups and thus reducing concurrent oligomerisation and polymerisation reactions^19^. Boric acid has also been used as a protective group for specific polysaccharides^18^.

We prepared mixtures of boric acid and choline chloride by mixing the dry components and heating the mixture while stirring at 80 °C. The first ratio to produce a visually transparent liquid was three parts of boric acid and five parts of choline chloride, even if the lowest melting point was received when two parts of boric acid was mixed with three parts of choline chloride. The 3:5 mixture was chosen for the fractionation experiments in order to limit the amount of boric acid to as low as possible due to possible concerns in its use.

Sawdust made from softwood was chosen as the raw material in our fractionation experiments, due to its high industrial availability for laboratory and bench-scale experiments. As lignocellulosic raw materials, softwoods are generally considered to be difficult to hydrolyse, primarily owing to the nature and amount of lignin involved. Chemical composition of hemicelluloses differs between different wood species; generally softwoods have lower xylose content and higher mannose content^20^. In addition, lignin in softwoods differs from that found in hardwoods and grasses. Softwood lignin consists almost exclusively of guaiacyl units, while hardwood and grasses also have syringyl units in their lignin structures. Guaiacyl units are more likely to C–C cross-link at the C-5 position of the ring – such cross-links can be formed during lignification as well as during delignification. In addition, because these C–C cross-links cannot be hydrolysed by acid or base, the delignification of softwoods requires harsher conditions than for hardwood and grasses^21^. Sawdust (referred hereon to as coarse) was used in the experiments as such. In addition, Wiley-milling was used to refine a comparative sawdust sample with a 1 mm particle size (referred to as fine) to create more active surface for the fractionation experiments. Because sawdust naturally contains 30–40% water, a sample was also prepared for comparison by drying sawdust in a vacuum at 140 °C to achieve a total water content of 2%.

**Figure 1:**
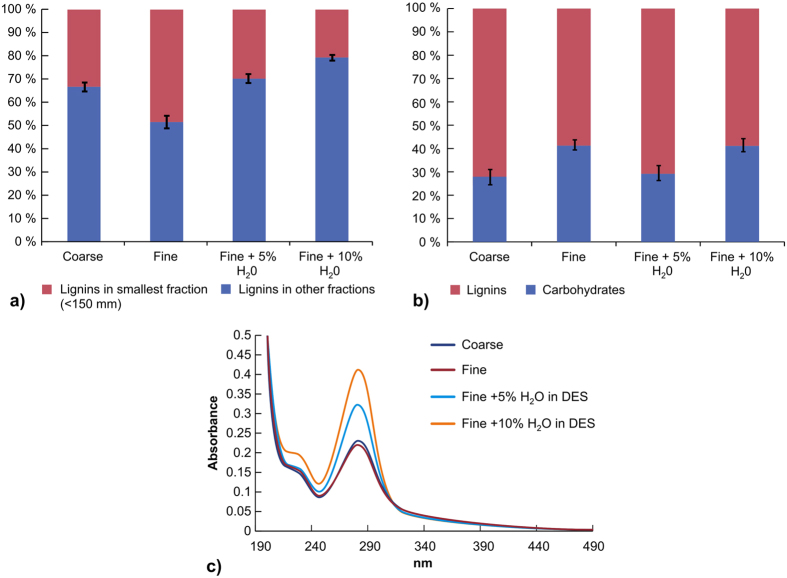
Percentage distributions of fractions after treatment at 95 °C.

Sawdust samples were treated with the boric acid/choline chloride low melting composition at 95 °C. For comparison, samples were fractionated also by diluting the low melting mixture with 5% and 10% of water to lower the viscosity of the mixture. The low melting mixture was stirred with the sawdust overnight and the mixture was then diluted with boiling water and passed through a series of metallic sieves (1 mm, 0.3 mm and 0.15 mm). The mixture was then separated into four fractions, the smallest fraction having a particle size of <0.15 mm. The particle sizes of each fraction and thus the fractionation efficiency of the system were confirmed using a particle size analyser. The relative amounts of the various fractions received based on different starting materials, as well as the fractionation equipment set-up, are presented in Fig. 1. The largest share of the <0.15 mm fraction was obtained for the fine sample, whereas the yields of the smaller fractions fell notably when the material was completely dried at 140 °C. This could result from thermally induced cross-linking of wood components that can significantly weaken its solubility in the low melting mixture system and furthermore decrease the porosity of wood. Additionally, drying the sawdust halts the penetration of the low melting mixture into the material and decreases the yield, but this is often the procedure for industrial use of wood.

**Figure 2:**
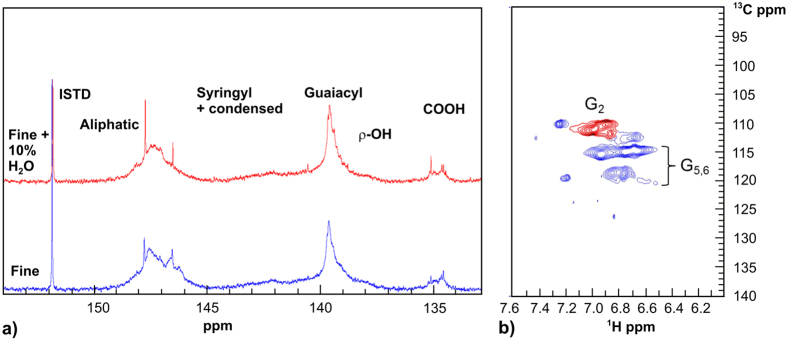
(**a)** Relative portions of lignin content in the smallest fraction <0.15 mm and the three larger fractions with standard deviations. The relative portions of lignin in the smallest fraction are between 20 and 49%. (**b)** Carbohydrate and lignin contents in the smallest fraction <0.15 mm with standard deviations. In all cases, the relative fractions of lignin are over 55%. (**c)** UV-VIS spectra of the solutions acquired from <0.15 mm fractions. The signal at 283 nm indicates the presence of furans (5-hydroxymethylfurfural (5-HMF), furfural).

The amount of lignin in each fraction was quantified by treating the fractions with sulphuric acid in order to hydrolyse the polysaccharides, followed by filtration and a gravimetric analysis of the lignin particles, as well as an analysis of the monosaccharide composition of the solution using high-performance anion-exchange chromatography with pulsed amperometric detection (HPAEC/PAD)^22^. The solubilised lignin remaining in the solution (<2%) was quantised using UV-visible spectrophotometry. Figure 2a shows the compositions of all fractions and the individual yields of the overall lignin content of different starting materials in the smallest fraction. The relative portions of lignin in the smallest fraction <0.15 mm and in the other three fractions are shown in Fig. 2b.

Lignin was the major component in all of the <0.15 mm fractions. The fine material provided the highest yield for the <0.15 mm fraction as well as the highest amount of total lignin fractionated from wood (Table 1). The smaller particle size of the fine sawdust clearly enables the low melting mixture to penetrate the wood more effectively, resulting in the highest overall yield of lignin, but more polysaccharides are also fractionated. The purity of the lignin was slightly higher when sawdust was used as such, or when 5% water was added to the low melting mixture.

UV-VIS measurements of the low melting solution remaining after the precipitation of lignin revealed the presence of 5-hydroxymethyl (5-HMF) and furfural among the degradation products, which were otherwise mainly organic acids (formic acid, acetic acid and levulinic acid) (Fig. 2c). Capillary electrophoresis (CE)^23^ was used for the quantitative analysis of these compounds. In a comparison of the composition of degradation products with and without the addition of water, the highest yield was achieved when 10% water was added to the mixture. The overall yield of furans and organic acids corresponded to 27% of the theoretical maximum yield achievable from the total hemicellulose content (see Supplementary Table S6), but this amount takes no account of the glucose created by the hydrolysis of glucomannans (the general composition of softwood is thought to be cellulose 41.7%, lignin 27.4% and hemicelluloses 28.3%, of which glucomannan accounted for 16.3%, glucuronoxylan 8.6% and other polysaccharides 3.4%^24^. The addition of water clearly improves the hydrolytic cleavage of hemicelluloses, but decreases the fractionation efficiency of the low melting mixture. The effect of water on the composition and properties of DESs has been studied in detail by several authors, and it has shown that the gradual addition of water to DES mixtures slowly causes the solvent properties to disappear, but even up to 50% of water can be added without losing all properties that are considered advantageous^11^. It is presumed that the glucose detected during a carbohydrate analysis (Table 1) originated in glucomannan-type hemicelluloses present in the wood, but could also partly originate from low molecular weight fractions of cellulose. However, the low amount of glucose residues in the smallest fraction implies that cellulose is more stable and therefore not extensively solubilised by the low melting composition. The hydrolysis of hemicelluloses could play an important part in the fractionation mechanism of lignin using the low melting composition.

**Figure 3:**
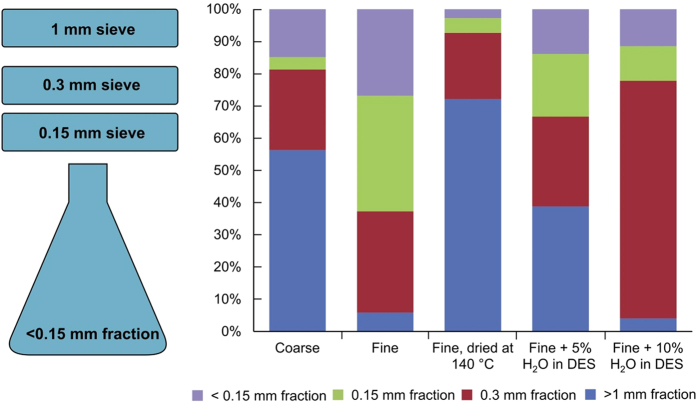
(**a)** Compiled ^31^P NMR spectra of two samples, Fine and Fine +10% H_2_O < 0.15 mm fractions in the low melting mixture. Kraft lignin (softwood) was measured as a reference. (**b)** Aromatic/unsaturated (δC/δH 90−155/6.0−8.0) regions in the 2D HSQC NMR spectra of the lignin extracted with the low melting mixture. See Supplementary Table S4. for signal assignments. Internal standard was marked by ISTD.

The chemical composition of lignin was characterised by ^31^P NMR (Fig. 3a), in accordance with the method developed by Granata *et al*.^25^. Characterisation revealed that the condensed units were largely missing from the NMR spectrum of the lignin extracted with the low melting mixture. A specific calculation of various functionalities (Fig. 3b) also revealed that the phenolic units formed as a result of cleavage of the aryl ether linkages during known fractionation methods are low in quantity, which also indicates a softer fractionation mechanism. The most typical lignin inter-unit linkages were further quantified using 2D NMR, in accordance with the method developed by del Rio *et al*.^26^, (Table 2). The main inter-unit linkages detected were the alkyl-aryl ethers (β–O–4′), followed by resinols (β–β′) and phenylcoumarans (β–5′), which were the only structural units present in the lignin. β–O–4′ linkages are generally considered to be the main indication of the severity of the fractionation process, and most current process development is aimed at preserving these structures8. The quantity of β–O–4′ linkages is high, close to that reported for wheat straw^26^ and higher than that reported for any wood-based lignins^27^.

The molecular weight of DES-lignin was determined using size-exclusion chromatography. The DES-lignin exhibited a weight-average (Mw) molecular weight of 3,273 g mol^−1^ and a number-average (Mn) molecular weight of 2,454. Additionally, the DES-lignin exhibited narrow polydispersity, with a Mw/Mn of 1.33, further indicating that the lignin is close to what is commonly believed to be its natural molecular weight^9^.

**Figure 4:**
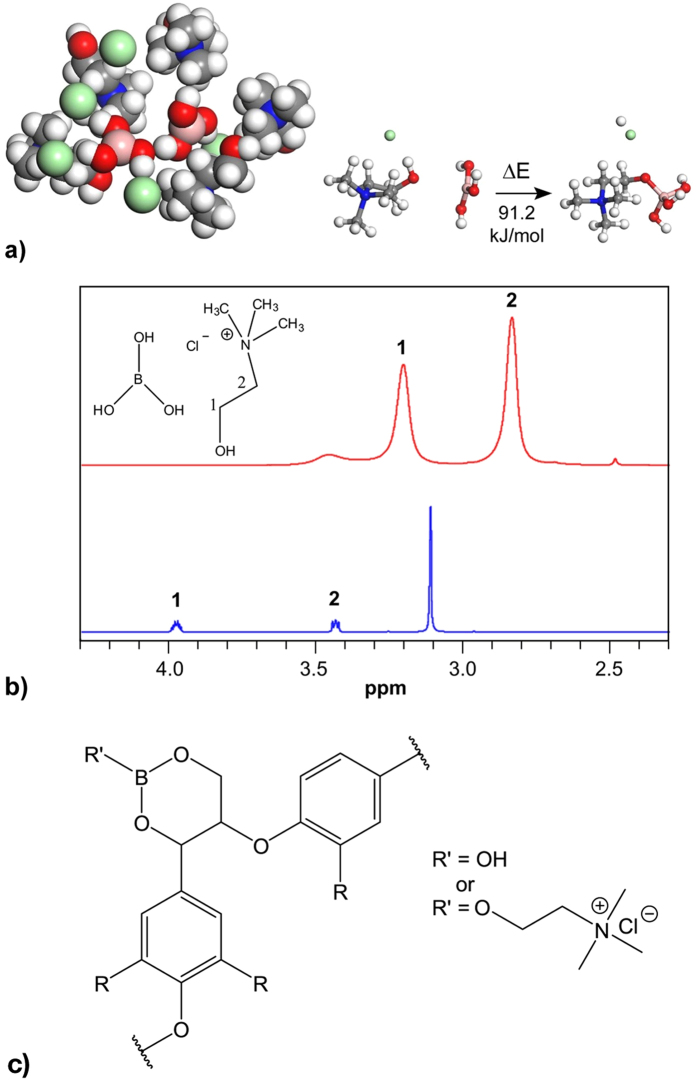
(**a)** Example of coordination between choline chloride and boric acid at 298 K, and complex formation reaction between choline chloride and boric acid. Light red: boron. Grey: carbon. Green: chlorine. White: hydrogen. Blue: nitrogen. Red: oxygen. (**b**) ^1^H-NMR specra of choline chloride in D_2_O (below) and DES mixture prepared from boric acid-d3 and choline chloride-(trimethyl-d9) (above). Quaternary ammonium hydrogens of choline chloride and hydrogens of boric acid are deuterated and thus absent from the upper spectrum. Chemical shifts have been referenced to tetramethylsilane. (**c)** Proposed bonding mode of the low melting components and lignin during fractionation.

Molecular modelling is a commonly applied technique for investigating the chemical reactions and properties of solid materials and polymers. Molecular dynamics (MD) simulations were performed in order to understand the extent of bonding that occurs between choline chloride and boric acid and the reason for the stability of the formed low melting mixture. MD simulations indicated that ionic and hydrogen bonding between choline chloride and boric acid can be very effective; the distance between the hydroxyl group of choline chloride and the hydroxyl groups of boric acid varied from 1.8 Å to 2.1 Å. The probability for these short distances was almost 2.5 times higher at 398 K than 298 K. Regardless of the temperature of the mixture, the distance between the chlorine ion and the hydroxyl groups of boric acid varied from 1.9 Å to 2.4 Å. However, the probability of bonding was notably smaller (~50%) when compared to the probability of bonding between boric acid and choline chloride at 398 K. When bonding between the components (Fig. 4a) was modelled, it was observed that the reaction is endothermic and requires energy (91.2 kJ/mol) to proceed. Interestingly, a strong initial decrease in the temperature was also noted in the preparation of the low melting mixture when the mixture was heated vigorously. The ^1^H-NMR spectra of the low melting composition was acquired by preparing a low melting mixture with deuterated components to avoid using any external solvents or components and thus receiving a spectrum of the low melting composition without interference. The spectra of the low melting mixture and pure choline chloride in D_2_O (Fig. 4b.) do not allow extensive comparisons to be made between the chemical shifts of choline chloride in either solvent due to the influence of the solvent on the values of chemical shifts. However, no new peaks appeared in the NMR spectrum of the low melting mixture, further supporting the stability of the components in the mixture and the proposed bonding mechanism.

Boric acid is capable of forming borate esters with lignin^19^. By forming covalent or hydrogen bonds, boric acid can hinder reactions (e.g. condensation reactions) through protection of the hydroxyl groups. In addition, role of non-covalent interactions can be essential. Reactions between choline chloride and boric acid in organic solvents or as low melting mixtures are not described in the literature, but in aqueous solutions they mainly exist as separate choline cation and various borate anions^28^. On the other hand, chelated orthoborate anions did not display any significant reactivity towards choline cations either in specific ionic liquids systems^29^, indicating that choline reactivity with borate anions may be energetically less favourable than that of various diols, triols and hydroxyl acids, which can form hydrolytically stable borate chelates even in purely aqueous conditions^30^. At elevated temperatures, boric acid can catalyse carbohydrate isomerisation and subsequent dehydrative conversions into furfurals and carboxylic acids^31^. Recently, this has also been demonstrated in DES systems^32^. It can be presumed that, to some extent, a similar phenomenon also occurs in boric acid: the choline chloride low melting mixture may play a major role in the detachment of lignin from other wood components. From the opposite perspective, boric acid prevented cellulose hydrolytic degradation in sulfolane^33^ via the formation of protective boric acid chelate on the cellulose surface. As boric acid and choline chloride are incapable of fractionating wood when applied as individual components, the formation of a low melting mixture with high stability and its subsequent interaction with lignin via a protective, direct interaction, are highly possible. The proposed covalent interaction between lignin and the low melting mixture has been presented in Fig. 4c.

## Discussion

Since most pulping methods have been devoted to producing high-quality cellulose, lignin is often disregarded and its use has so far been limited. Whether the aim is to use lignin in its polymeric form or as a source of valuable aromatic monomers, most known fractionation methods lead to a significant decrease in ß-O-4 units and a loss of native-type lignin structures^8^,^9^. Because the yields of most aromatic depolymerisation products – such as syringyl, guaiacyl and p-hydroxyphenyl aromatic products – correlate closely with the quantity of ß-O-4 linkages present in the original lignin, these linkages should therefore be preserved. The removal of hemicellulose from cellulosic fractions has required further treatment and increased the overall costs of biorefining processes. New, preferably simultaneous strategies without the need for several process steps are therefore required for the efficient separation of components. The fractionation method based on a low melting mixture of boric acid and choline chloride provides a new cost-efficient and low-risk opportunity to use components derived from wood as a sustainable source of chemicals.

## Additional Information

**How to cite this article**: Hiltunen, J. *et al*. Using a low melting solvent mixture to extract value from wood biomass. *Sci. Rep*. **6**, 32420; doi: 10.1038/srep32420 (2016).

## Supplementary Material

Supplementary Information

## Figures and Tables

**Table 1:** Monosaccharide composition of the smallest (<0.15 mm) fraction^a^.

Sample	RHA	ARA	GAL	GLU	XYL	MAN	Total lignin content in the <150 mm fraction (%)^a^	Share of the total lignin content in wood (%)^b^			
Spruce	**0.17**	**1.1**	**1.9**	46	**5.4**	**12**	28.6				
Coarse	**<0.1**	**0.1**	**0.1**	21	**0.5**	**1.3**	68.8	33.3			
Fine	**<0.1**	**0.2**	**0.3**	38	**1.4**	**3.2**	55.5	49.5			
Fine +5% H_2_O	**<0.1**	**0.1**	**0.2**	27	**1.2**	**2.8**	68.5	29.9			
Fine +10% H_2_O	**<0.1**	**0.1**	**0.1**	39	**1.2**	**2.9**	56.1	20.7			

^a^Monosaccharide results are presented as mg/100 mg of dry matter (monosaccharides of hemicelluloses are highlighted in bold).

^b^The lignin content of the smallest fraction and how that corresponds to the total content of lignin present in wood.

**Table 2:** Content of most typical lignin inter-unit linkages per 100 C9 unit determined by 2D NMR, and the lignin functionalities determined by ^31^P NMR^a^.

Sample	β-O-4	β–β	β-5	Aliphatic OH	Carboxylic acid	Condensed + Syringyl	Guaiacyl	Catechols	p-OH-phenyl	Phenolic OH	Total OH
Fine	67	22	11	1.61	0.22	0.53	1.08	0.00	0.15	1.76	3.59
Fine + 10 wt-% H_2_O	63	20	10	1.43	0.23	0.54	1.24	0.00	0.11	1.89	3.55
Kraft^27^	2.4	1.5	0.1	1.6	0.6	3.3	0.9	0.0	0.0	4.2	5.8

^a^mmol/g lignin, calculated according to the lignin content of the samples, while the effect of carbohydrates on aliphatic hydroxyls was not taken into account.
