# Supplementary material for: Using a low melting solvent mixture to extract value from wood biomass
Source: Sci Rep. 2016 Sep 7;6:32420. doi: 10.1038/srep32420 (PMC5013283; doi:10.1038/srep32420)
Supplement: Supplementary Information [file srep32420-s1.doc]

SUPPLEMENTARY INFORMATION

Using a low melting solvent mixture to extract value from wood biomass

Jaakko Hiltunen, Lauri Kuutti, Stella Rovio, Eini Puhakka, Tommi Virtanen, Taina Ohra-Aho, and Sauli Vuoti*

VTT Technical Research Centre of Finland, Biologinkuja 7, P.O. Box 1000, FI-02044 VTT, Finland * Corresponding author: e-mail address: sauli.vuoti@gmail.com

**Table of Contents**

**I. General Considerations S1**

**II. Preparation of the Low melting Solvents S2**

**III. Characterization of the Low melting Mixtures**  **S2**

IV. Fractionation of Softwood with Low melting Solvents S3

**V. DSC S3**

**VI. Py-GC/FID/MS S4**

**VII. ^13^C CP/MAS NMR S6**

**VIII. 1D and 2D NMR Characterization of Low Melting Mixtures and Lignin S7**

**IX. ^31^P NMR Measurement**s **S10**

**X. Microscopy Images S10**

**XI. Particle Size Distributions S11**

**XII. FT-IR S11**

**XIII. Molar Mass Distribution S12**

**XIV. 5-HMF and Furfural Production S12**

**XV. Molecular Modelling S13**

**XVI. Mass Balances S15**

**XVII. References S16**

**I. General Considerations**

All commercially available compounds were purchased and used as received, unless otherwise noted. Softwood saw dust (spruce) was used in fractionation experiments. Softwood saw dust (“coarse”) was collected from a Finnish saw mill and had an average particle size of 1-2 mm. A portion of the coarse material was ground using Wiley-mill equipped with a 1.0 mm sieve and labelled as “fine”. A sample was also prepared from fine by drying the grinded sawdust in oven at 140 °C for 4 hours prior to fractionation experiments. The moisture content of the sawdust feedstock was determined by Karl-Fischer titration after drying at 105 °C overnight. All the components used in preparation of the low melting mixtures were purchased from Sigma (St. Louis, MO, USA) and dried before use. Sodium hydroxide and hydrochloric acid were purchased from Merck. Infrared Spectroscopy (IR) was used to monitor structural changes of both undissolved and dissolved solid fractions. IR spectra were measured using Nicolet iS50 FT-IR equipped with Omnic software and an ATR sampling accessory (Thermo Fisher Scientific Inc., Rockford, USA). The IR spectra (4000–400 cm^-1^) were recorded with a resolution of 4 cm^-1^ and 32 scans per sample. Samples were hydrolyzed with sulphuric acid to determine their monosaccharide compositions, and the monosaccharides were analysed with HPAEC/PAD with pulse amperometric detection (Dionex ICS 3000LC) equipped with CarboPac PA1 column. Unsolubilized lignin concentration was determined gravimetrically, and solubilized lignin was quantized by UV-visible spectrophotometry. The 5-hydroxymethyl furfural (5-HMF), furfural, formic acid, acetic acid and levulinic acid contents in the low melting solutions were analyzed with a P/ACE MDQ capillary electrophoresis instrument equipped with a photodiode array UV/Vis detector (PDA) (Beckman-Coulter, Inc., Fullerton, CA, USA). Prior to the acid analyses, chloride was removed with OnGuard II Ba/Ag/H cartridges (Dionex Ltd.) UV-VIS spectrophotometer (Perkin Elmer UV/VIS/NIR Spectrometer Lambda 900) equipped with a 1 cm path length cell was used for the qualitative analysis of the acidic low melting liquors containing the furfurals and organic acids. Absorbance within 190-600 nm was measured using 1 nm spectral resolution. Liquid samples were diluted 1:100 v/v with ultrapure water. Samples were referenced to ultrapure water. ^31^P NMR was used for chemical characterization of lignin. Microscopic images were recorded with an Olympus BH-2 transmission light microscope without staining. For composition analyses, the lignin was hydrolysed^1^, and the acid insoluble Klason lignin was quantified gravimetrically. Acid soluble lignin was determined spectroscopically at 205 nm (TAPPI UM250 um‐83, 1991). The carbohydrate content of hydrolysate was analysed by borate anion‐exchange chromatography with post‐column derivatisation and detection at 560 nm^2^. The ash content was gravimetrically determined after incineration at 800 °C. The complete oxidation method was used for elemental analysis on a CE Instruments CHNS Flash 1112 Analyzer system. Methoxyl groups were determined according to Vieböck et al.^3^.

**II. Preparation of the low melting solvents**

The low melting solvent mixtures were prepared as reported previously^4^ by thoroughly mixing boric acid and choline chloride and heating the mixture under stirring at 80 °C. The first ratio to produce a transparent liquid with a practical viscosity level at this temperature was 3:5 (proportion of boric acid given first). When the amount of boric acid was lower than three parts, a small amount of water was needed to form a transparent low melting mixture.

**III. Characterization of the low melting Mixtures**

Boric acid and choline chloride form a transparent, viscous liquid when mixed in molar ratios 3:5-1:1 (proportion of boric acid given first) at elevated temperatures. Viscosity values for the solutions in different temperatures have been presented in Table S1. When a 1:3 ratio mixture was prepared, 4% of water by weight was needed to form a transparent liquid. The viscosity of this mixture was slightly higher than for the 3:5 mixture. Addition of water to the 3:5 mixture lowered the viscosity of the mixture slightly, but had no clear impact in its appearance and no precipitation was seen. Wood naturally contains water, so the low melting mixture is presumed to be combined with water during fractionation processes.

pH measurements in deep eutectic solvents or low melting mixtures are not conclusive or absolutely comparable because of the unspecified liquid junction potential (LJP) set at the solvent interface^5^. However, their measurement may give valuable data for comparing different molar ratios of mixtures and their observed acidities in biomass mixtures containing water. pH values for different low melting mixtures have been presented in Table S2. The acidity was increased when the amount of boric acid was increased. From the theoretical perspective, this could primarily be due to increased complex formation between choline chloride and boric acid, which simultaneously releases hydrochloric acid, but hydrogen bond acidity could also have an impact. It has been described that the hydrogen bond acidities in a specific DES system can be associated with factors such as molecular ratio of the components, water content, and temperature. Furthermore, an inverse relationship between the acidity and the temperature may exist ^6,7^. Similar effects could also be observed in the boric acid – choline chloride low melting system. The apparent acidity of the mixture was decreased by the addition of water, possibly by suppression of interactions that facilitate acidity.

**Table S1.** Viscosity values at different temperatures.

| **Mixture** | **70 °C** | **80 °C** | **90 °C** | **100 °C** | **120 °C** |
| --- | --- | --- | --- | --- | --- |
| **1:3 (4% H_2_O)** | 210 | 151 | 93 | 60 | 43 |
| **3:5** | 152 | 96 | 67 | 48 | 29 |
| **3:5 (4% H_2_O)** | 144 | 92 | 63 | 46 | 29 |
| **1:1** | 133 | 89 | 62 | 44 | 28 |

**Table S2.** pH values at different temperatures.

| **Mixture** | **70 °C** | **80 °C** | **90 °C** | **100 °C** |
| --- | --- | --- | --- | --- |
| **1:3 (4% H2O)** | 1.2 | 1.36 | 1.70 | 1.74 |
| **3:5** | 0.46 | 0.67 | 0.94 | 1.16 |
| **3:5 (4% H_2_O)** | 0.78 | 0.88 | 1.01 | 1.27 |
| **1:1** | n/a | n/a | n/a | n/a |

**IV. Fractionation of Softwood with Low melting Solvents**

The fractionation experiments were carried out using a Tornado Plus Overhead Mechanical Stirring System with 6 seat reactor carousel manufactured by Radleys (Radleys Discovery Technologies, UK). The inside temperatures in the vessels were 95 °C and 110 °C in the fractionation trials. The stirring speed of PTFE centrifugal stirrer paddles was to 100 rpm and reaction time was adjusted to 16 hours. The load of lignocellulosic material was 5 wt% in the low melting solutions. The low melting solutions were prepared using dry salts, which were first mixed and then heated up to the reaction temperature. The choline chloride-boric acid mixture formed a clear solution at approximately 60 °C. Sawdust was as added slowly under mechanical stirring to ensure homogeneous mixing of the solution and sawdust. After the mixing was completed, water (temperature 70 °C) was added to the biomass mixture consisting of the low melting composition and sawdust to obtain an aqueous mixture. The aqueous biomass mixtures were then fractionated using a set of three metallic sieves (1 mm. 0.3 mm and 0.15 mm) and the fractions collected from the sieves. The smallest (<0.15 mm) fraction that was passed through the three sieves was collected to an Erlenmeyer flask. The different fractions were washed with 400 ml of boiling water. The separated fractions were then dried at 50 °C overnight and weighed. The under 0.15 mm fraction was filtered while hot and washed with 100 ml of cold water to remove the residual low melting components, and finally dried at 50 °C overnight and weighed. The aqueous low melting solution was then evaporated under vacuum at 150 °C for several hours and then lyophilized.

**V. DSC Measurements**

The melting temperatures (Tm) of low melting mixture samples were determined by Mettler Toledo Differential Scanning Calorimeter model DSC820 system STARe SW 12.00 (Mettler Toledo GmbH, Switzerland). N_2_ flow rate 50 ml min^-1^ and 40 ml sealed aluminum crucibles were used. Temperature profile with following steps was used: 1) first heating phase from -80 °C to 100 °C, 2) cooling phase to 80 °C and 3) second heating phase from -80 °C to 100 °C. Heating and cooling rate of 10 °C/min was used in all cases. The exact DSC values have been presented in Table S3. Tm measurements of the low melting mixtures show that the melting point of the low melting mixture was already lowered remarkably at a ratio of 1:3 in comparison with the individual components. The melting point of the mixture sets at around 46-47 °C starting around the ratio of 3:5.

**Table S3.** DSC values for the low melting solvents.

| **Ratio** | **Tm^1^ (°C)** | **ΔCp  (J g^-1^ K^-1^)** | **Tm^2^ (°C)** | **ΔCp  (J g^-1^ K^-1^)** |
| --- | --- | --- | --- | --- |
| 1:3 (+4% H_2_O) | 23.0  68.8 | 20.1  3.7 | 23.1  69.0 | 26.8  8.0 |
| 3:5 | 23.6  46.0 | 7.1  6.25 | 25.3  47.9 | 3.6  8.2 |
| 1:1 | 47.4 | 21.3 | 46.6 | 36.3 |

**VI. Py-GC/FID/MS**

The Py-GC/FID/MS measurements were performed with a filament pulse pyrolyzer (Pyrola2000, PyrolAB, Sweden), which was connected to a gas chromatograph (Agilent 7890B) with a flame ionization detector (FID) and a mass spectrometer (Agilent 5977A). Pyrolysis products obtained at 580 °C were separated using a capillary column (J&W, DB-1701, 30 m x 0.25 mm, film 1 µm), using the following temperature program: initial temperature 100 °C, held for 1 min, rate of increase 4 °C/min to 265 °C and held for 17.75 min. Constant flow rate of 1.0 ml (He) was used through the first column. For the dual detection with FID and MSD degradation products were split in ratio of 1:1 via three-way splitter kit (Agilent G3440B). Two inert fused silica columns one linked to FID (1.1 m x 0.18 mm i.d.) and other to MSD (2.9 m x 0.18 mm i.d.) using outlet pressure of 3.8 psi was used. FID was operated at 300 °C using: H2 flow: 45 ml/min; air flow: 450 ml/min: make up (He) 50 ml/min. Mass spectra were obtained in the full scan mode using mass range of 46-800 m/z (EI, 70 eV). Interface and ion source temperatures were 250 and 230 °C, respectively. Mass spectra of main degradation products were identified using data from the literature^8^ and commercial NIST2012 library. GC/FID gas chromatograms were used to data evaluation due to the similar responses of the compounds obtained by FID.

The composition of lignin and carbohydrates in coarse and fine were analysed without pre-treatment by Py-GC/FID. The pyrograms of the samples are shown in Fig. S1. Main pyrolysis degradation products of lignin (numbers) and carbohydrates (letters) are marked in pyrogram and the identities are presented below the Fig. S1 *p*-Hydroxyphenyl (H) and guaiacyl type (G) lignin degradation products, typical for softwood lignin, were detected from both samples in which the (G) type structures were dominant. Intensity of oxygen containing (G) type degradation products (peak numbers 13-23), which are typical structures originating from native wood lignin^9^, was higher for fine than coarse. This indicates that the structure of the coarse lignin was altered more during treatment than the fine lignin. Main degradation products of carbohydrates originated from cellulose (b, d, e, g) and hemicelluloses (xylose (a, f) and arabinose (c)). Intensity of the carbohydrate degradation products was slightly higher in the coarse than the fine sample, except for arabinose.


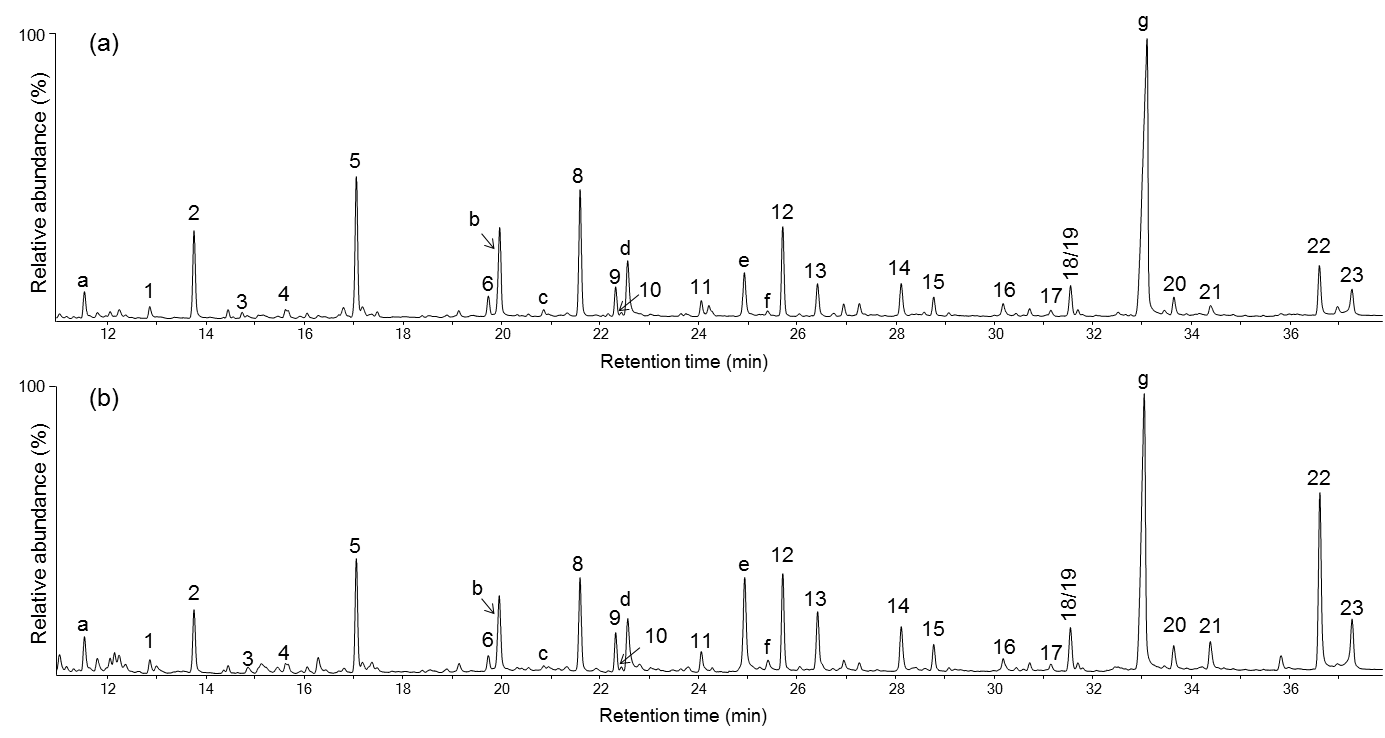


**Fig. S1.** Py-GC/FID chromatograms of coarse under 0.15 mm (a) and fine under 0.15 mm (b). Peak identities of lignin derivatives (origin): 1. phenol (H); 2. guaiacol (G); 3. 2-methylphenol (G); 4. 4-methylphenol (H); 5. 4-methylguaiacol (G); 6. 4-ethylguaiacol (G); 7. 4-vinylphenol (P); 8. 4-vinylguaiacol (G); 9. eugenol (G); 10. 4-propylguaiacol (G); 11. *cis*-isoeugenol (G); 12. *trans*-isoeugenol (G); 13. vanillin (G); 14. guaiacylpropyne (G); 15. guaiacylallene (G); 16. homovanillin (G); 17. acetoguaiacone (G); 18. guaiacylacetone (G) ; 19. propioguaiacone (G); 20. 4-(oxy-allyl)guaiacol (G); 21. 4-(1-hydroxy-prop-2-enyl)guaiacol (G); 22. dihydroconiferyl alcohol (G); 23. *cis*-coniferyl alcohol (G); 24. *trans*-coniferyl alcohol (G); 25. coniferaldehyde (G).

Peak identities of carbohydrate derivatives (origin): a 4-hydroxy-5,6-dihydro-(2H)-pyran-2-one (xylose); b 5-hydroxymethyl-2-tetrahydofuraldehyde-3-one (glucose); c 1,5-anhydroarabino­furanose (arabinose); d 5-hydroxymethyl-2-furaldehyde (glucose); e 1.4-dideoxy-D-glycero-hex-1-enopyranos-3-ulose (glucose) f 1,4-anhydroxylopyranose (xylose); g 1,6- anhydro­glucopyranose (glucose).

**VII. ^13^C CP/MAS NMR Measurements**

The ^13^C CP/MAS NMR measurements were performed with an Agilent 600 NMR spectrometer with magnetic flux density of 14.1 T using a 3.2 mm triple-resonance MAS NMR probe. For all samples, 20000 transients were accumulated using a 2 ms contact time and a 3 s recycle time with a MAS rate of 10 kHz. In all experiments a proton decoupling of 80 kHz was used. The ^13^C CP/MAS NMR measurements were used to get a rough view on the proportion of lignin and carbohydrates in each fraction and further support the general observations of the fractionation efficiency. According to Figures S2-S4, the amount of lignin (>100 ppm regions) were clearly higher in the smaller fraction.

**
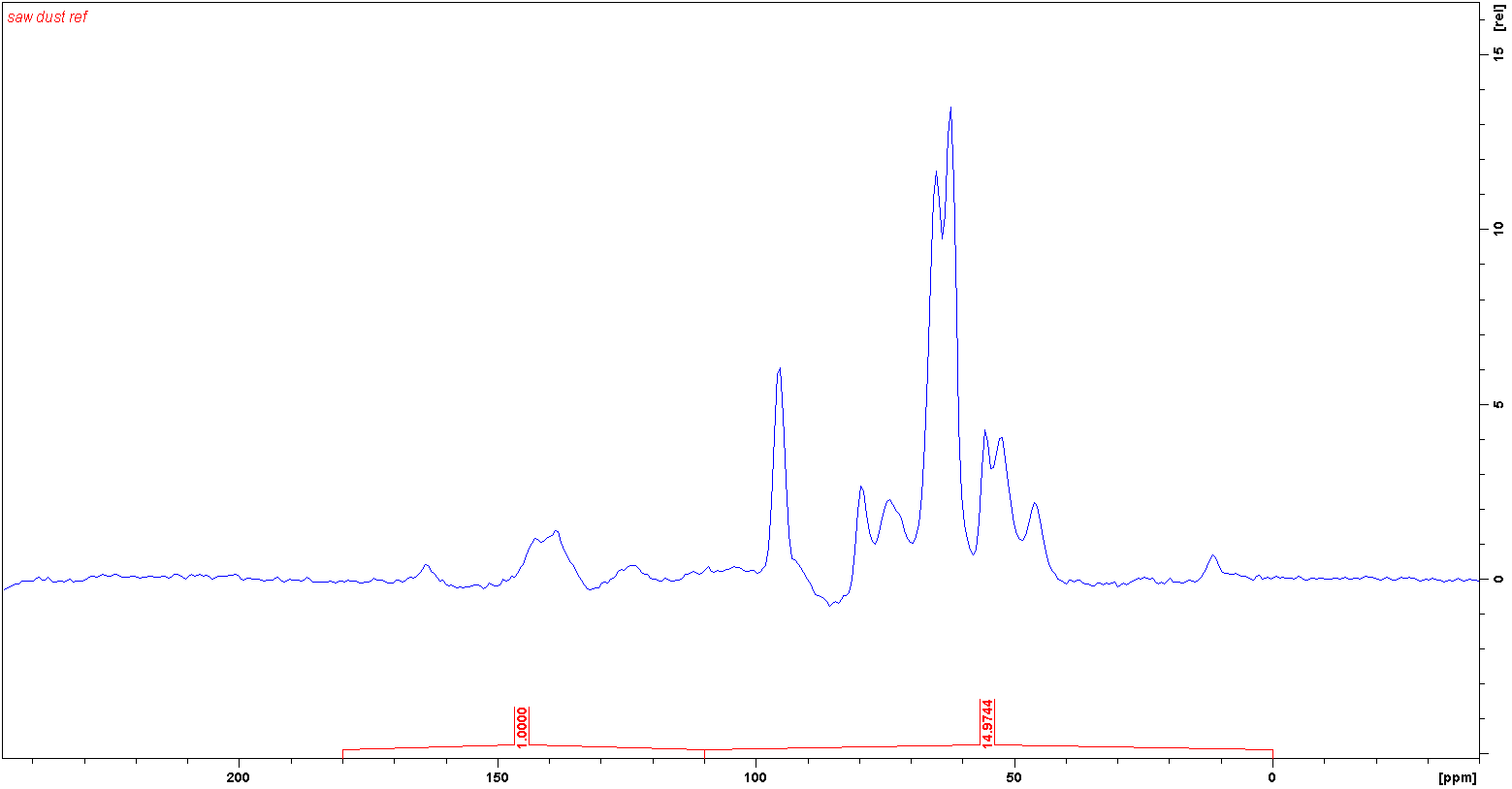
**

**Fig. S2.** Raw material (spruce saw dust)

**
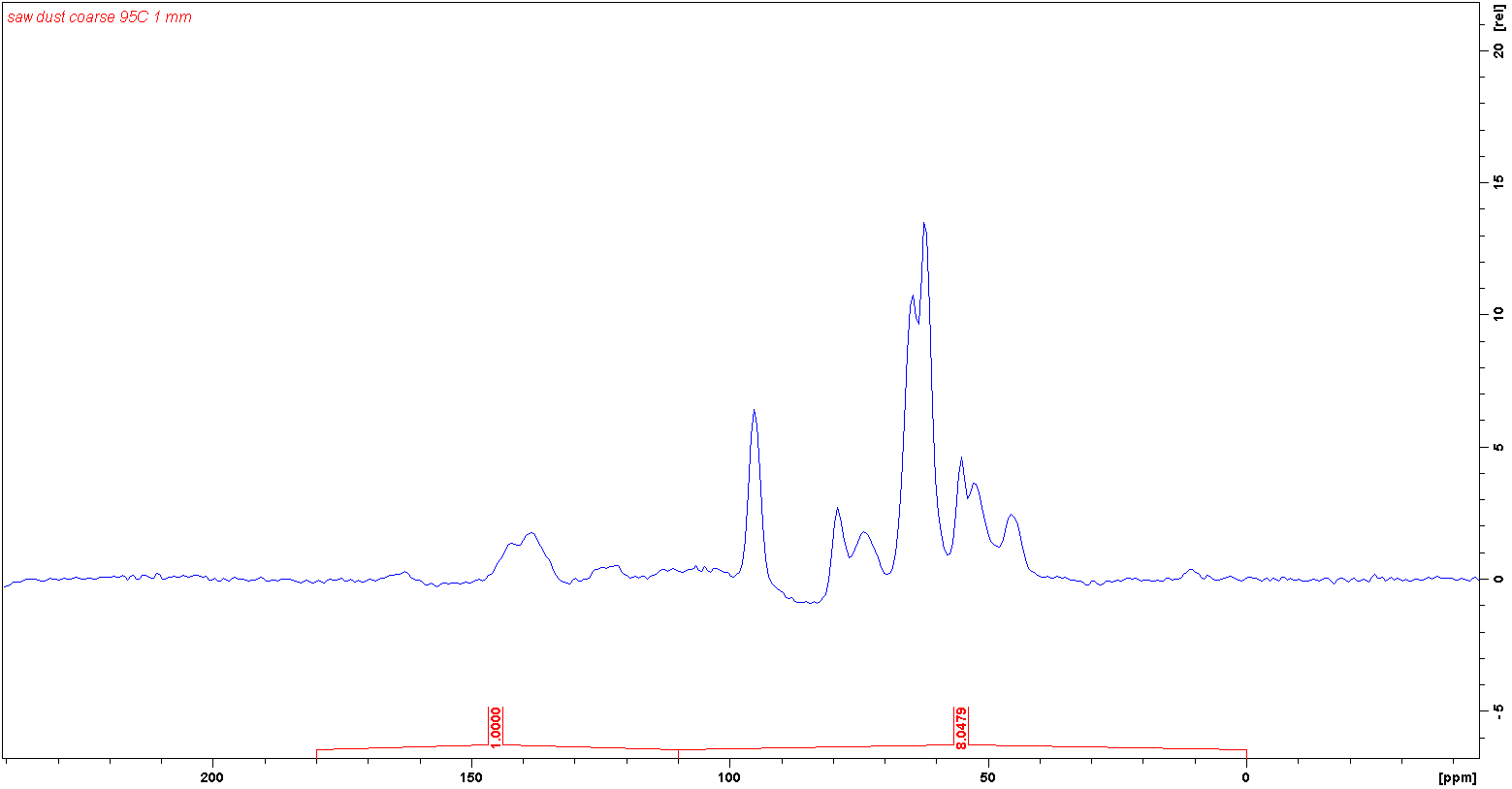
**

**Fig. S3.** Saw dust coarse 95 °C 1 mm fraction.

**
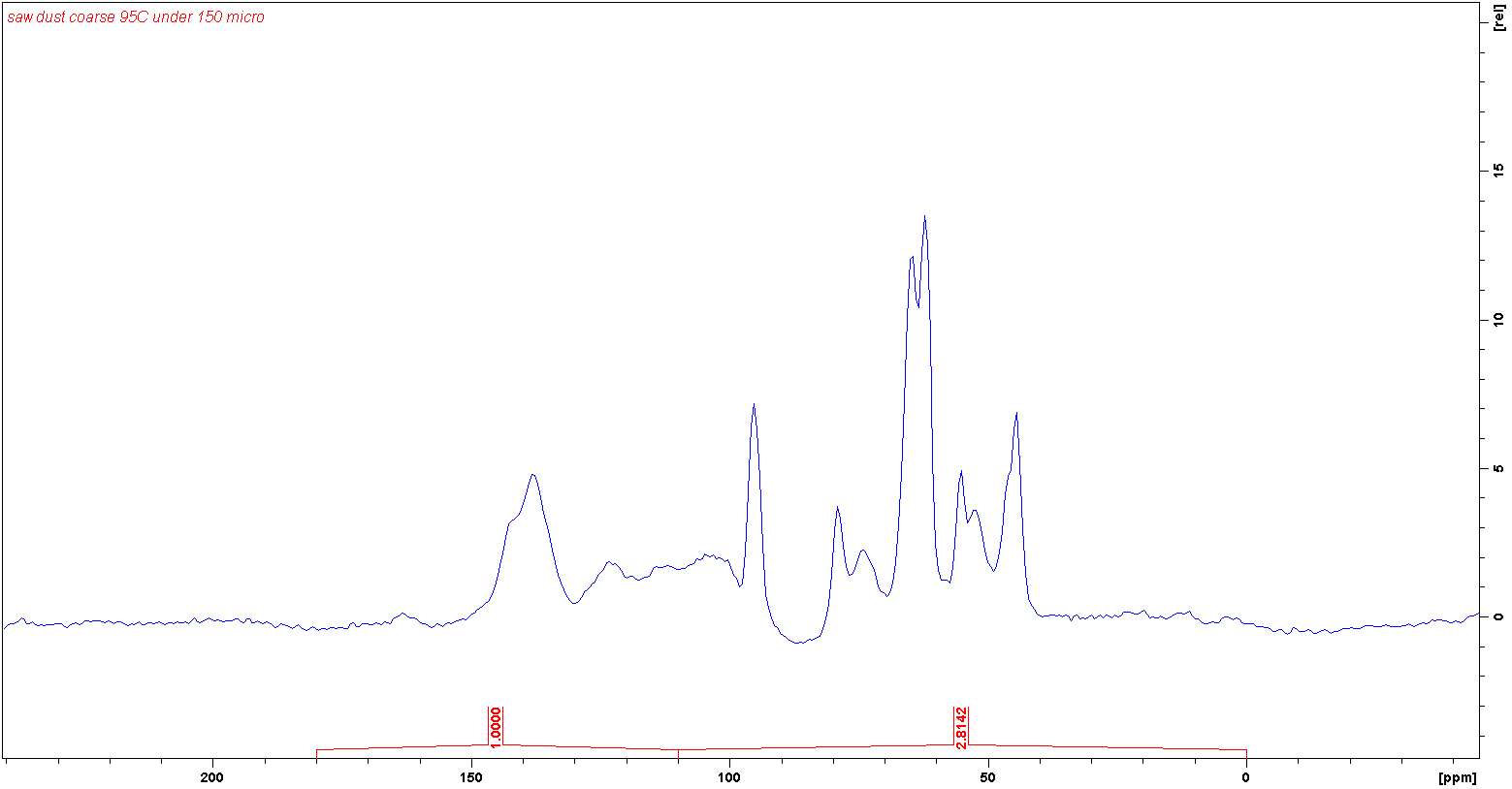
**

**Fig. S4.** Saw dust coarse 95 °C under 0.15 mm fraction.

**VIII. 1D and 2D NMR characterization of the low melting mixture and lignin using liquid state NMR**

The deuterated low melting mixture used for the NMR characterization (main text Fig. 4b) was prepared similarly as the standard low melting mixtures described in section II of the supplementary. Boric acid-d3 and choline chloride-(trimethyl-d9) were used for the mixture in order to enable locking of the spectrometer without the addition of external solvents, and thus studying the hydrogen bonding of the low melting mixture without interference from external components. The signals were referenced to TMS (0.01 % v/v). The full scale spectrum of the deuterated 3:5 boric acid - choline chloride low melting mixture is shown in Figure S5.


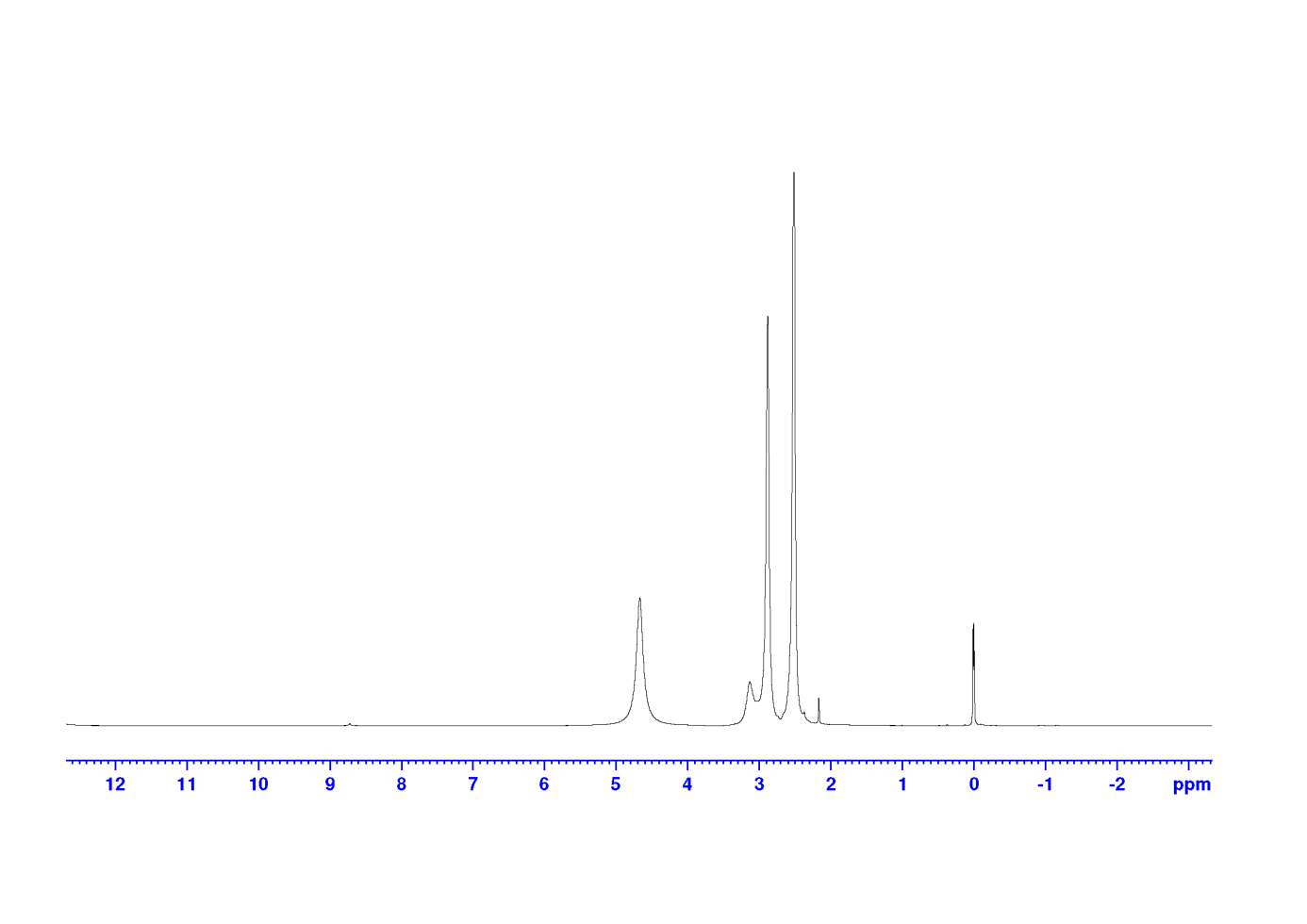

Fig. S5. ^1^H NMR spectrum of the deuterated 3:5 low melting mixture. The peak of TMS is shown at 0 ppm, H_2_O at ~ 4.65 ppm.

The 2D-NMR assisted quantitation of the relative abundances of lignin substructures β-O-4', β-5' and β-β' is based on volume integration of the signals representative to those structures observed in a two dimensional heteronuclear single quantum coherence (HSQC) NMR spectrum^10^ (see Fig. 2b in the main article and Fig. S5 and Table S4). 2D HSQC NMR spectra were recorded at 30 ºC with a Bruker AVANCE III 600 MHz spectrometer, equipped with a cryogenically-cooled 5 mm TCI gradient probe with inverse geometry, and using a sensitivity enhanced pulse sequence that utilizes adiabatic pulses on carbon channel (hsqcetgpsisp2.2). Lignin samples were dissolved (40mg/0.75 mL) in DMSO-d6. Spectral widths of 12 ppm (from -1 to 11 ppm) and 240 ppm (from 5 to 245 ppm) for the ^1^H- and ^13^C-dimensions were used, and 145 Hz as an average value for J-coupling between proton and carbon over one bond. The number of transients was 64 for 256 time increments. Prior Fourier transformation the data in direct dimension was zero-filled (one-fold), and for the indirectly detected dimension a forward linear prediction was used. Quadratic shifted sinebell window functions were used for both dimensions as window functions.

The semiquantitative analysis of the correlation peaks was performed using Bruker TopSpin 3.1 software. The lignin inter-unit linkages based on Cα–Hα correlations were calculated as described previously, and also assignments of the lignin signals are based on that given in literature^10-12^. The relative amount of the structural units is calculated from the corresponding volume integrals using the C_2_-H_2_ signal from guaiacyl units (G_2_) as an internal reference. In the aromatic spectral region it is noteworthy that only signals from guaiacyl units appear in the spectrum.

**Table S4.** Assignments of the 2D HSQC spectrum correlation signals. The labelled signals were used in calculation of the relative amounts of the subunits β-O-4’, β-5’ and β-β’. Peak picking was performed by TopSpin 3.5.

| **δ_c_ / δ_H_ (ppm)** | **Assignment** | **Label in spectrum** |
| --- | --- | --- |
| 53.1 / 3.44 | C_β_-H_β_ in phenylcoumaran substructures |  |
| 55.4 / 3.37. 52.9 / 3.06 | -OCH_3_ in methoxyl groups |  |
| 59.8 / 3.58. 3.40 | C_γ_-H_γ_ in γ-hydroxylated β-O-4’ substructures |  |
| 62.5 / 3.69 | C_γ_-H_γ_ in phenylcoumaran substructures |  |
| 65.9 – 76.2 / 3.00 – 3.83 | Hemicelluloses |  |
| 70.8 / 4.13. 3.72 | C_γ_-H_γ_ in β-β' resinol substructures |  |
| 71.1 / 4.74 | C_α_-H_α_ in β-O-4' substructures linked to a guaiacyl (**G)** unit | β-O-4' |
| 80.9 / 4.60 | C_β_-H_β_ in γ-acylated β-O-4' substructures linked to a **G** unit |  |
| 83.8 / 4.29 | C_β_-H_β_ in β-O-4' substructures linked to a guaiacyl (**G)** unit |  |
| 86.6 / 5.45 | C_α_-H_α_ in phenylcoumaran substructures | β-5' |
| 85.0 / 4.61 | C_α_-H_α_ in β-β' resinol substructures | β-β ' |
| 110.2 / 7.24 | C_2_-H_2_ in oxidized α-ketone **G** units |  |
| 111.2 / 6.99 | C_2_-H_2_ in **G** units | G_2_ |
| 117.2 – 121.2 / 6.49 – 7.26 | C_5_-H_5_. C_6_-H_6_ in **G** units | G_5.6_ |


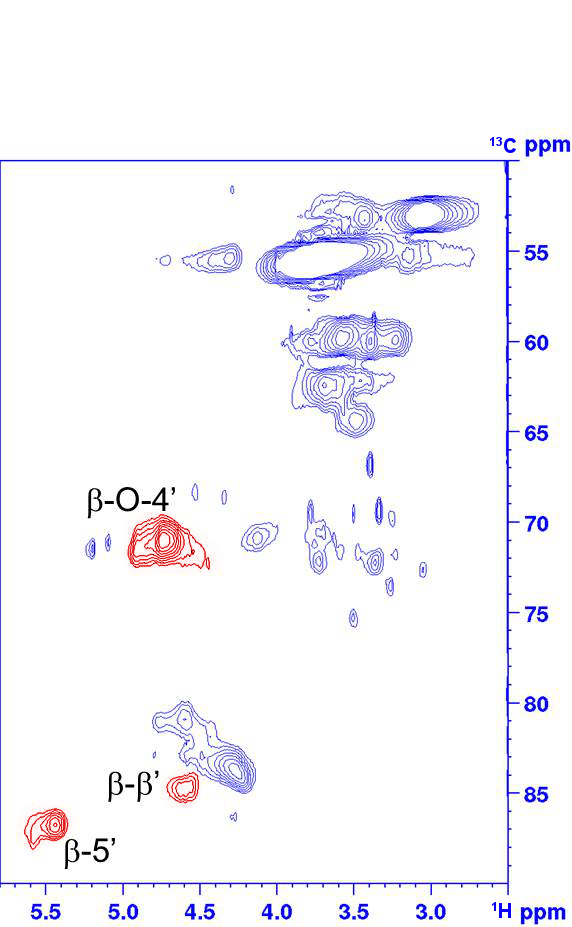


**Fig. S5.** Side chain region of a 2D HSQC spectrum. The integrated regions are shown in red. The relative amounts of the lignin inter-unit linkages β-O-4’, β-β’, and β-5’ were calculated from the volume integrals of the signals as marked on the spectrum. Assignments can be found from the Table S4.

**IX. ^31^P NMR Measurements**

The ^31^P NMR measurements were performed immediately after sample preparation at room temperature with Bruker Avance 500 MHz NMR spectrometer equipped with a BBI probehead, and using a 30° excitation pulse and 512 scans with 5 s delay between successive scans. For the ^31^P NMR analyses^13^, each lignin sample was accurately weighted (25 mg) and dissolved in N.N-dimethylformamide (300 µL) in 10 mL vial. After partial dissolution, pyridine (100 µL), internal standard solution (ISTD) (200 µL) *endo*-N-Hydroxy-5-norbornene-2.3-dicarboximide (e-HNDI. 0.005 mmol) in pyridine/CDCl_3_ (1.6/1. v/v) and Cr(acac)_3_ solution (50 µL) (11.4 mg/1 mL) in pyridine/CDCl_3_ (1.6/1. v/v) were added. Then, phosphitylation reagent (150 µL) 2-chloro-4.4.5.5-tetramethyl-1.3.2-dioxaphopholane [P.R.(II)] was added drop-wise. Finally, CDCl_3_ (300 µL) was added to the solution and clear brown to black solution was achieved (unless otherwise stated below in results section). Dissolution was improved by heating (45°C), vortexing and continuous stirring with magnetic stirrer for several hours. Chemical shifts are reported relative to the sharp signal (132.2 ppm) originating from the reaction between water and P.R. II.

**X. Microscopy Images**

Microscopic pictures of the under 0.15 mm fractions of coarse and fine showed that the smallest precipitate consisted of flocky particles (See Fig. S6a). When the material was dried at 140 ºC, the particles were different in shape, resembling sand particles and therefore labelled as sandy (See Fig. S6b). Typically the flocky particles were acquired as co-precipitates, i.e. after the biomass was solubilized in the low melting mixture and then precipitated, whereas the sandy particles resembled native wood particles.


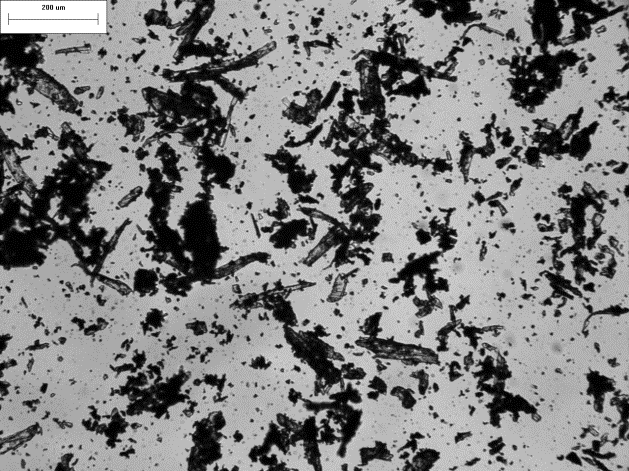

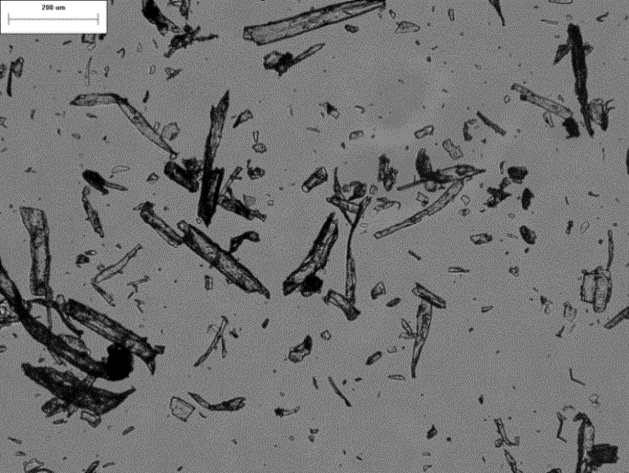


**Fig. S6**. Image comparison of flocky type precipitate (A: fine. Co-precipitation, scale bar 0,2 mm) and sandy type precipitate (B: fine. dried at 140 °C. particle precipitation, scale bar 0,2 mm).

**XI. Particle Size Distributions**

The particle size distributions revealed that the fractionation process using metallic sieves were very effective to fractionate the process samples. The mean values of the selected samples were between 27,4 to 108,9 micro meters (Table 5) . The particle sizes were measured with Beckman Coulter LS 13 320 Particle Size Analyzer.

**Table 5.** Particle size distributions of coarse and fine fractions after fractionation at 95 º C.

| **Fraction** | **Mean** | **10 %** | **25 %** | **50 %** | **75 %** | **90 %** |
| --- | --- | --- | --- | --- | --- | --- |
| Coarse < 0.15 mm | 51.4 | 9.7 | 20.5 | 49.1 | 112.7 | 332.2 |
| Fine 0.15 mm | 108.9 | 26.0 | 59.4 | 128.0 | 245.5 | 406.8 |
| Fine < 0.15 mm | 27.4 | 8.2 | 14.3 | 24.2 | 51.2 | 117.6 |

**XII. FT-IR Analysis**

FT-IR spectra of the different fractions acquired after treating the sawdust at 95 °C showed that spectra in general were very similar regardless of the wood meal used in the experiments. Fig. S7 shows the FTIR spectra obtained from the different fractions. The signals associated with lignin structures at 1263-1265 cm^-1^, 1508-1510 cm^-1^ and 1596-1592 cm^-1^ are slightly enlarged in the smaller fractions, which indicates that the amount of polysaccharides has decreased, and therefore signals associated with lignin were more clearly seen. The signal at 3333-3338 cm^-1^ indicates O-H intramolecular stretching of cellulose, while the signal at 2875-2899 cm^-1^ represents the C-H stretching of cellulose. The lignin content in all fractions has increased remarkably according to the IR spectra, which is consistent with other characterization methods.


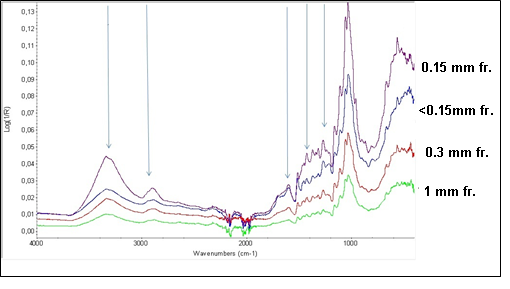


**Fig. S7.** FTIR spectra of the fractions acquired from the fine sample treated with the low melting mixture at 95 °C. Sample filtrated precipitate is under 0.15 mm fraction.

**XIII. Molar Mass Distribution**

The molar mass distributions of lignins were determined by size exclusion chromatography in 0.1 M NaOH using MCX 1000 and 100 000 columns with UV detection (280 nm) and polystyrene suphonate (Na-PSS) calibration. The detected molar masses are summarized in Table S6.

**Table S6. Molar mass distributions of the lignin isolated from the under 0.15 mm fractions.**

| **Fraction** | **Mn** | **Mw** | **Mz** | **MP** | **Polydispersity** |
| --- | --- | --- | --- | --- | --- |
| Coarse under 0.15 mm 95 °C | 2370 | 3345 | 4836 | 2932 | 1.41 |
| Coarse under 0.15 mm 110 °C | 2745 | 4631 | 8809 | 3077 | 1.69 |
| Fine under 0.15 mm 95 °C | 2454 | 3273 | 4371 | 2890 | 1.33 |
| Fine under 0.15 mm 110 °C | 2579 | 4607 | 9374 | 3041 | 1.79 |
|  |  |  |  |  |  |

**XIV. 5-Hydroxymethylfurfural and Furfural Production**

Capillary electrophoresis (CE) was used for the quantitative analysis of 5-hydroxymethylfurfural, furfural and the organic acids in the low melting mixtures. This was done for the experiments carried out for the 95 °C and 110 °C experiments. The apparent high acidity of the low melting mixture could promote the formation of 5-HMF from hexoses and in smaller extent also the formation of furfural from pentoses of hemicellulose structures. The higher water content (10 %) improve the furan yields. The yield of 5-HMF in 10 % water content was the highest 29.1 mg/g of dry wood and 5% water content gave almost equal 5-HMF conversion. This is not yet an economically reasonable amount to collect, but by optimizing fractionation conditions and by applying biphase collection system there, the extraction of furans can be improved. If more than 10% water was added to the low melting mixture, the furan yield was decreased. Surprisingly, the 110 °C fractionation temperature was detrimental to the production of 5-HMF, as can be seen in Table S7. On the other hand, the yield of furfural consistently increased when the temperature was increased. The degradation reactions of both 5-HMF and furfural produce formic, acetic and levulinic acids. Consequently the yield of levulinic acid at 95 °C was rather low, but as expected, when the temperature was increased to 110 °C, the yield was notably increased. When the sawdust was dried at 140 °C, all yields were the lowest, indicating that drying produces wood meal, which is resistant to low melting mixtures at all temperatures (not shown in table S7).

**Table S7.** 5-HMF, furfural, formic acid, acetic acid and levulinic acid in the low melting mixtures in the 95 °C and 110 °C experiments in mg/g of dry wood.

| Fraction | 5-HMF | | Furfural | | Formic acid | | Acetic acid | | Levulinic acid | |
| --- | --- | --- | --- | --- | --- | --- | --- | --- | --- | --- |
|  | **95°C** | **110°C** | **95°C** | **110°C** | **95°C** | **110°C** | **95°C** | **110°C** | **95°C** | **110°C** |
| Coarse | 9.2 | 11.2 | 3.4 | 6.4 | 15.3 | 3.8 | 3.7 | 1.9 | 1.1 | -- |
| Fine | 10.5 | 8.8 | 4.2 | 5.5 | 16.6 | 4.1 | 4.4 | 3.0 | 1.4 | 3.9 |
| Fine + 5 wt-% H_2_O | 26.9 | 9.9 | 8.6 | 9.0 | 14.0 | 3.9 | 4.7 | 3.3 | 0.57 | 4.0 |
| Fine + 10 wt% H_2_O | 29.1 | 21.8 | 8.3 | 15.9 | 13.9 | 7.2 | 4.4 | 5.9 | 0.60 | 5.2 |

Dehydration of hexose or pentose by three water molecules forms 5-HMF and furfural (theoretical yield is respectively 70.0% and 63.3%)

Hexose - 3 x Water -> 5-HMF Pentose – 3 x Water -> Furfural

C_6_H_12_O_6_ – 3 H_2_O -> C_6_H_6_O_3_ C_5_H_10_O_5_ – 3 H_2_O -> C_5_H_4_O_2_

In spruce the hemicelluloses are mostly galactoglucomannans (15-20% of dry content of wood) and arabinoglucuronoxylans (5-10% of the dry content of wood). Thus, the theoretical values are between 15-20% x 0.7 = 10.5-14.0% for 5-HMF and 5-10% x 0.63 = 3.2-6.3% for furfural in regard to dry wood.

**XV. Molecular modelling**

Molecular dynamics (MD) simulations were performed for amorphous cell structures with Materials Studio software of BIOVIA^14^ with the *ab initio* based COMPASS forcefield (parameters derived based on *ab initio* data), which contains parameters for most of organic compounds. However, the COMPASS does not contain parameters for boron. Therefore, for boron containing mixtures, Dreiding forcefield was used, where general force constants and geometry parameters are based on simple hybridization rules rather than on specific combinations of atoms. Based on our comparative simulations with the COMPASS and Dreiding forcefields, getting in order between the compounds does not depend on the forcefields in this investigated system. The MD simulations were performed at 298 K, 323 K, 348 K and 398 K for choline chloride:boric acid mixture with and without water in the mixture. Simulation time was 100 ps and time step was 0.5 fs in the constant volume/constant temperature MD simulations. The investigated molar ratios were 3:1 and 5:3 and the density of mixture was 1.2 g/cm^3^. After the simulations, radial distribution functions were analyzed. This gave information about the coordination chemistry of compounds in the mixtures, e.g. the probability of hydrogen bonding between compounds forming the mixture.

Complex formation reaction between a hydrogen bond donor (choline chloride) and a hydrogen bond acceptor (boric acid) was investigated using density functional methods. Calculations were performed with the DMol^3^ Version 7.0 code implemented into Materials Studio version 7.0. In these calculations, the total electronic energy and overall electronic density distribution were solved in order to define the energetically stable structures for the hydrogen bond donor and the hydrogen bond acceptor, and compounds forming during the complex formation reaction^15^. Both in the geometry optimization of reagents and products, and reaction mechanism calculations, the exchange-correlation potential were described with gradient-corrected functional GGA-PW91. As a compromise between the accuracy and computational time of calculations, the semi-core pseudo­potentials were used for each element. These potentials replace core electrons by a single effective potential, reducing the computational cost, and they also introduce some degree of relativistic correction into the core. The atomic orbital basis set was described by the DNP (Double Numerical plus Polarization) basis set. This basis set includes a polarization p-function on all hydrogen atoms in order to describe hydrogen bonding as accurately as possible.

Based on our experimental tests for different low melting solvent combinations, the choline chloride – boric acid 5:3 mixture was detected to be a promising mixture for selective dissolution and fractionation of wood components. In order to estimate the probability for hydrogen bonding between choline chloride and boric acid compounds, MD simulations were performed for choline chloride – boric acid mixtures as molar ratios 3:1 and 5:3. After that radial distribution functions of the simulated mixtures were analysed (Fig. S8). It was detected that the closest distance of a chloride ion of choline chloride to boric acid is only about 0.2 nm. This is significantly a shorter distance than a distance (about 0.3 nm) between a chloride ion and a hydroxyl group of choline chloride. Because the distance between a hydroxyl group of choline chloride and boric acid is also short, 0.17 nm – 0.24 nm, this means that there is rather effective ionic and hydrogen bonding between the compounds.


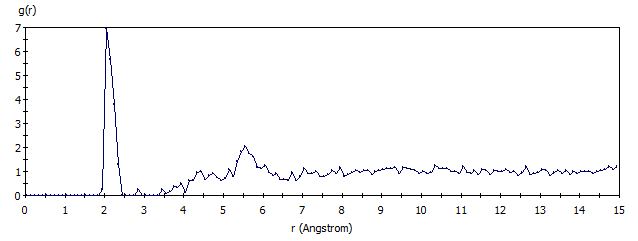


**Cl^-^ – OH**

**Fig. S8** Choline chloride:boric acid mixture at 298 K: Radial distribution functions for Cl^-^ – OH interactions.

In addition to these ionic and hydrogen bonding between different compounds, boric acid tends to form hydrogen bonded dimeric structures in the mixtures. In the dimer, there are two OH∙∙∙O interactions, and the strength of the hydrogen bonds can correspond even the strength of hydrogen bonding in water^16^. At the room temperature in the 3:1 mixture, the boric acid dimer with its hydroxyl groups can bond to even five choline chloride molecules via their chloride ions (Fig. S9). At the higher temperatures (323 K, 348 K and 398 K), interactions are weaker because of the more active motion of molecules.


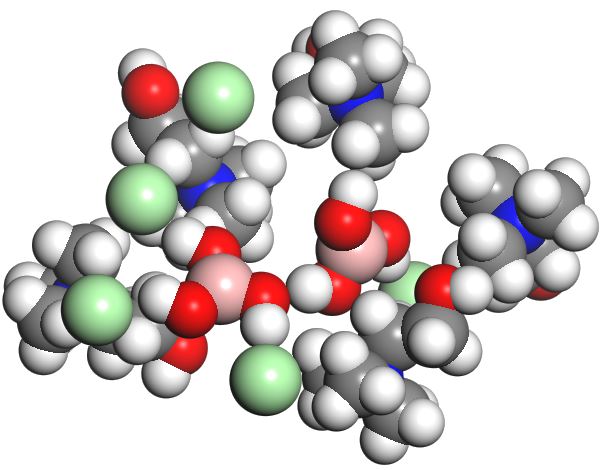


**Fig. S9**. Example of coordination between choline chloride and boric acid at 298 K. Light red: boron. Grey: carbon. Green: chlorine. White: hydrogen. Blue: nitrogen. Red: oxygen.

**XVI. Mass Balances**

The mass balance of all fractions has been presented in Table S8. In all cases the saw dust input was 2.7 grams accounting only the dry matter.

**Table. S8.** Mass balances in fractionation process as dry matter (unit grams).

| **Fraction** | **Coarse** | **Fine** | **Fine + 5% H_2_0** | **Fine + 10% H_2_0** |
| --- | --- | --- | --- | --- |
| 1 mm | 1.21 | 0.14 | 0.87 | 0.09 |
| 0.3 mm | 0.54 | 0.68 | 0.62 | 1.69 |
| 0.15 mm | 0.09 | 0.84 | 0.44 | 0.25 |
| under 0.15 mm | 0.34 | 0.62 | 0.31 | 0.26 |
|  |  |  |  |  |
| Total | 2.18 | 2.28 | 2.24 | 2.29 |
|  |  |  |  |  |

The carbohydrate and lignin contents of all fractions have been presented in Figure 10a (coarse) and b (fine).

a)
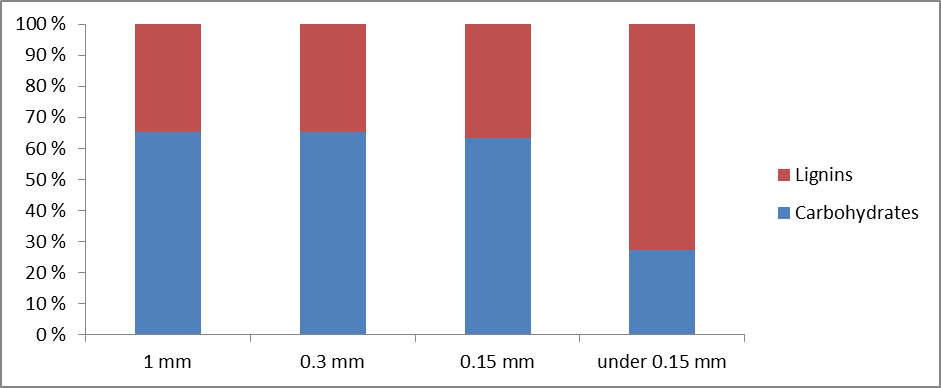


b)


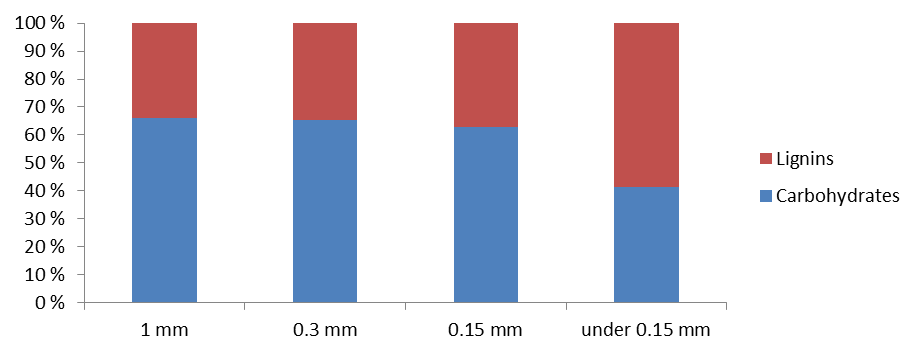


**Fig. S10.** The carbohydrate and lignin contents of all fractions a) coarse and b) fine.

**XVII. References**

1. Puls, J. Substrate analysis of forest and agricultural wastes. In: Biotechnology in agriculture; bioconversion of forest and agricultural plant residues. Ed. Saddler, J.N. CAB International, 13‐32. (1993).
2. Willför, S. et al. Carbohydrate analysis of plant materials with uronic acid‐containing polysaccharides – A comparison between different hydrolysis and subsequent chromatographic analytical techniques. Industrial crops and products, 29, 571‐580 (2009).
3. Vieböck, F., Schwappach, A. Eine neue Methode zur maßanalytischen Bestimmung der Methoxyl‐ und Äthoxylgruppe. I. Makro‐analyse. Berichte der deutschen chemischen Gesellschaft (A and B Series), 1930, 63, 2818–2823.
4. Hiltunen, J., Vuoti, S., Kuutti, L., Deep low melting solvents and their use WO 2015128550.
5. De Vreese, P. Electrochemical depositions in ionic liquids, Doctoral thesis, KU Leuven (2013).
6. Florindo. C. at al. Insights into the synthesis and properties of deep low melting solvents based on cholinium chloride and carboxylic acids. *ACS Sus. Chem. Eng*. **2**, 2416–2425 (2014).
7. Pandey, A., Pandey, S., Solvatochromic Probe Behavior within Choline Chloride-Based Deep Low melting Solvents: Effect of Temperature and Water, *J. Phys. Chem. B*, 118 (50), pp 14652–14661 (2014).
8. Ralph, J., Hatfield, R.D. Pyrolysis-GC-MS Characterization of Forage materials, J. Agric. Food Chem. 39, 1426-1437 (1991).
9. Ohra-aho, T., Tenkanen, T., Tamminen, T. Direct analysis of lignin and lignin-like components from softwood kraft pulp by different pyrolysis-GC/MS techniques, Journal of Analytical and Applied Pyrolysis 74, 123-128 (2005).
10. Del Río. J.C et al. Structural characterization of wheat straw lignin as revealed by analytical pyrolysis. 2D-NMR. and reductive cleavage methods. Journal of Agricultural and Food Chemistry 60, 5922-5935 (2012).
11. Sette. M., Wechselberger. R., Crestini. C. Elucidation of lignin structure by quantitative 2D NMR. Chemistry – A European Journal 17, 9529-9535 (2011).
12. Wen. J.-L., Sun. S.-L., Xue. B.-L., Sun. R.-C. Recent advances in characterization of lignin polymer by solution-state nuclear magnetic resonance (NMR) methodology. Materials 6, 359-391 (2013).
13. Granata, A., Arguropoulos, D.S. 2-Chloro-4,4,5,5-tetramethyl-1,3,2-dioxaphospholane, a Reagent for the Accurate Determination of the Uncondensed and Condensed Phenolic Moieties in Lignins. J. Agric. Food Chem. 43, 1538- 1544 (1995).
14. MS Modeling. Release 7.0, San Diego: Accelrys Software Inc. (2013).
15. Leach. A. R. Molecular Modelling, Principles and Applications, 2nd ed. (Pearson Education Limited. Essex. ( 2001).
16. Elango, M., Parthasarathi. R., Subramanian, V. & Sathyamurthy, N. Bowls. Balls, and Sheets of Boric Acid Clusters: The Role of Pentagon and Hexacon Motifs. J. Phys. Chem. A. 109, 8587-8593 (2005).
